# The immunosuppressive mechanisms induced by sepsis and the corresponding treatment strategies

**DOI:** 10.3389/fimmu.2025.1643194

**Published:** 2025-10-24

**Authors:** Qinfen Gao, Yajun Teng, Ling Zhu, Wei Zhang, Zengzheng Li

**Affiliations:** ^1^ Department of Emergency, The First People’s Hospital of Yunnan Province, The Affiliated Hospital of Kunming University of Science and Technology, Kunming, China; ^2^ Department of Infectious Diseases and Hepatology, The First People’s Hospital of Yunnan Province, The Affiliated Hospital of Kunming University of Science and Technology, Kunming, China; ^3^ Department of Critical Care Medicine, The First Affiliated Hospital of Kunming Medical College, Kunming, China; ^4^ Yunnan Province Clinical Research Center for Hematologic Disease, The First People’s Hospital of Yunnan Province, Kunming, China; ^5^ Yunnan Provincial Clinical Medical Center for Blood Diseases and Thrombosis Prevention and Treatment, The First People’s Hospital of Yunnan Province, Kunming, China; ^6^ Yunnan Atherosclerosis Cooperation Base of Chinese and Western Medicine, The First People’s Hospital of Yunnan Province, Kunming, China; ^7^ Department of Hematology, The First People’s Hospital of Yunnan Province, Affiliated Hospital of Kunming University of Science and Technology, Kunming, China

**Keywords:** sepsis, immunosuppression, immune metabolism, immune homeostasis, cytokines, therapeutic target

## Abstract

Sepsis is a life-threatening organ dysfunction caused by the dysregulation of the body’s response to infection. It is characterized by a high incidence, high mortality rate, and high medical burden, and is a major global health threat. Although updated treatment guidelines have reduced the mortality rate during the acute phase, survivors still face a high long-term risk of recurrent infection and death. Recent studies have shown that the long-term high mortality rate of sepsis is closely related to the immunosuppression it induces. Sepsis-induced immunosuppression originates from the disruption of immune homeostasis, characterized by excessive release of anti-inflammatory cytokines, increased apoptosis of immune cells (especially lymphocytes), T cell exhaustion, and expansion of immune regulatory cells (Tregs, MDSCs). Reduced expression of human leukocyte antigen-DR (HLA-DR) and upregulated expression of immune checkpoint molecules (PD-1, CTLA-4, TIM-3, etc.) further exacerbate immunosuppression. This article systematically reviews the immune imbalance state and related mechanisms of patients with sepsis, and summarizes new immunotherapy strategies such as immune stimulatory factors (GM-CSF, IL-7, IL-15), immune checkpoint inhibitors (anti-PD-1/PD-L1, anti-CTLA-4, anti-TIM-3), and emerging therapies (mesenchymal stem cells, calprotectin inhibitors, TREM-1 inhibitors). The aim is to enhance clinicians’ understanding of sepsis-induced immunosuppression, facilitate early intervention, and reduce the incidence and mortality of long-term complications.

## 1 Background

The 2021 European Society of Intensive Care Medicine/American Society of Intensive Care Medicine Sepsis Guidelines follow the 2016 version of the Sepsis 3.0 definition, which states that the dysregulation of the host response caused by infection leads to life-threatening organ dysfunction (1). Over the past few decades, timely use of antibiotics, fluid resuscitation, and organ support treatment have reduced the in-hospital acute mortality rate of sepsis, but the long-term mortality rate remains high. Survivors often require long-term medical support and remain in the ICU, consuming a large amount of resources (2, 3). According to the latest data from the Global Burden of Disease, there were nearly 50 million sepsis patients worldwide in 2017, with 11 million deaths (4), making it a major health challenge and social economic burden. Reports show that the mortality rate of sepsis survivors in the first year after discharge is approximately 15%, and the mortality rate in the following 5 years is 6-8% (5). Our team’s research has found that the mortality rate of sepsis patients is 27%, and those with residual organ dysfunction have a worse prognosis (6). Current evidence indicates that immune homeostasis imbalance is closely related to the occurrence and development of sepsis (7). Sepsis patients can simultaneously or sequentially present with excessive inflammatory response and immunosuppression. The former leads to early tissue damage and organ dysfunction; the latter, if persistent, is closely related to an increased risk of secondary infection, metabolic disorders, and poor long-term prognosis (7, 8).

**Figure 1:**
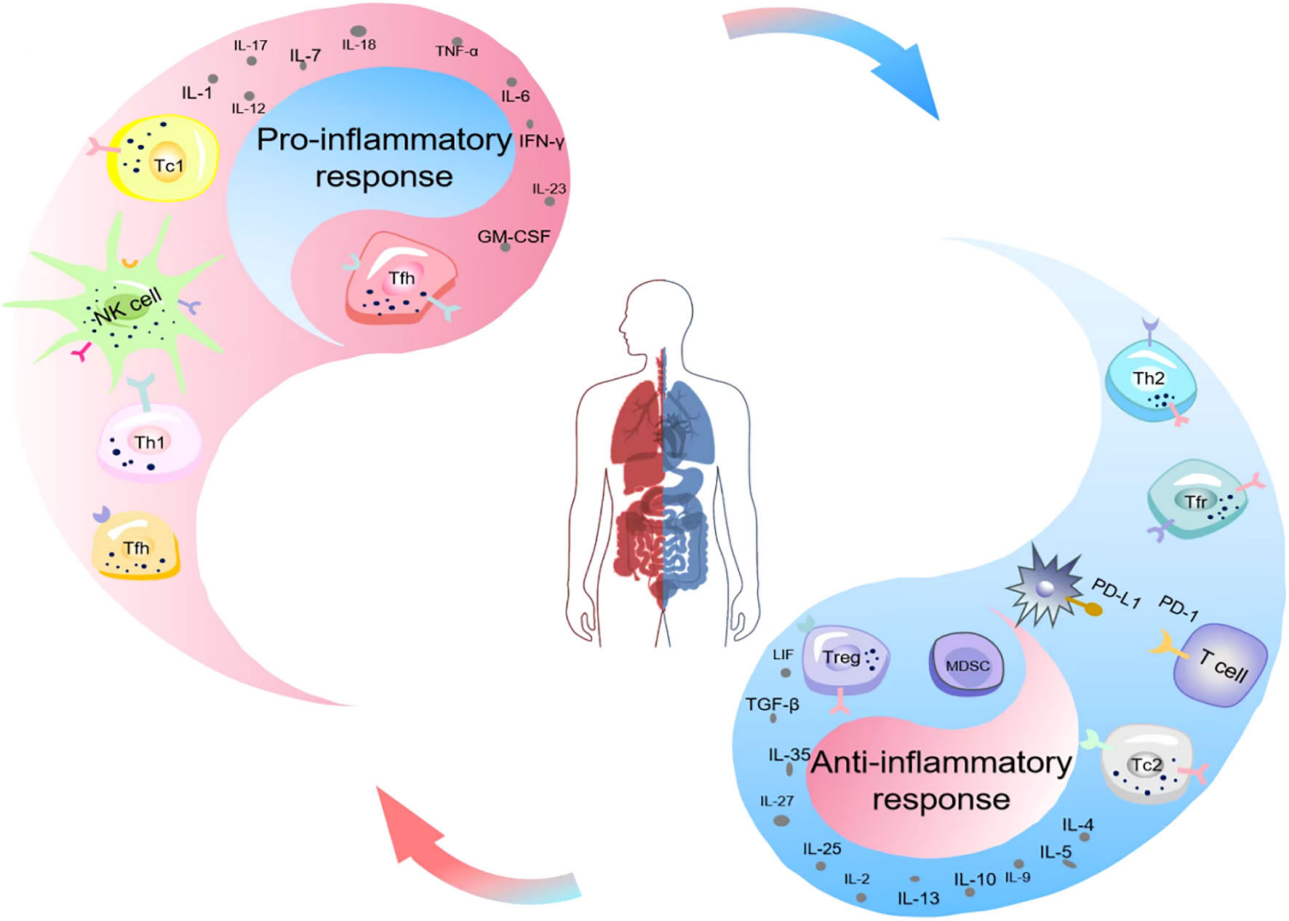
Schematic illustration of the imbalance of immune homeostasis (Yin-Yang imbalance) in sepsis. Ancient Chinese philosophers held that the generation of all things stemmed from the interaction between yin and yang. The interaction of yin and yang maintained the dynamic physiological balance of the human body, and the occurrence of diseases was often associated with the imbalance of yin and yang. The equilibrium between the pro-inflammatory response (yang) and the anti-inflammatory response (yin) of the organism sustained the immune homeostasis of the body. Nevertheless, the occurrence of sepsis disrupted this balance. The upregulated expression of pro-inflammatory cytokines released by inflammatory cells and the activation of the complement and coagulation systems gave rise to cytokine storms and MODS. Simultaneously or subsequently, immune cell death, the expansion of MDSCs and Tregs, the increased release of anti-inflammatory cytokines and immune checkpoint molecules, and the decreased expression of HLA-DR resulted in immunosuppression. MODS, Multiple Organ Dysfunction Syndrome; MDSCs, Myeloid-Derived Suppressor Cells; Treg, Regulatory T Cells; PD-1, Programmed Cell Death Protein 1.

Immune homeostasis plays a crucial role in the pathophysiology of sepsis and determines the clinical outcome (9). Immune suppression is caused by excessive release of anti-inflammatory cytokines and the decline in the number and function of immune cells (10, 11). During sepsis, inflammation and immune suppression may occur sequentially or simultaneously (12). In the early stage of systemic inflammatory response, if innate immunity promptly clears the pathogen, immune balance can be rapidly restored. If innate immunity fails to completely eliminate the infection, it will activate the immune response after recognizing pathogen-associated molecular patterns (PAMPs) and damage-associated molecular patterns (DAMPs), clear the pathogen and present antigens to adaptive immune cells. The upregulated expression of pro-inflammatory cytokines released by inflammatory cells and the activation of complement and coagulation systems lead to excessive inflammation, which subsequently causes cytokine storm and Multi-Organ Dysfunction Syndrome(MODS). At the same time or subsequently, the release of anti-inflammatory cytokines and immune checkpoint molecules increases, HLA-DR expression decreases, immune cell death and the expansion of regulatory cells lead to immune suppression, increasing the susceptibility to secondary infections, which is the main reason for the poor prognosis of sepsis patients (7, 12). In recent years, studies on sepsis have gradually revealed its complex mechanisms in inducing immune suppression (**Figure 1**), and a deeper understanding of these mechanisms is crucial for improving the diagnosis and treatment levels of sepsis.

Studies have shown that merely controlling the early inflammatory storm is insufficient to correct the long-term immune disorder in sepsis. Immune regulation therapy has become the focus of research, aiming to restore immune balance. Currently, emerging therapies such as immune stimulatory factors, checkpoint inhibitors, and mesenchymal stem cells have shown potential in improving immune disorders and organ functions (13). This article systematically summarizes the research progress on the mechanism of immune suppression induced by sepsis, covering aspects such as damage to immune effector cells, expansion of immune regulatory cells, release of anti-inflammatory cytokines, expression of immune checkpoint molecules, metabolic disorders, persistent inflammation, downregulation of HLA-DR, ferroptosis, and autophagy, and focuses on discussing new treatment strategies, providing references for the precise staging and effective treatment of sepsis.

## 2 The mechanism of immune suppression induced by sepsis

### 2.1 Immune cell damage and exhaustion

The high mortality rate and long-term complications of sepsis are closely related to immune cell damage (14, 15). The immunosuppressive state of sepsis is accompanied by the depletion and increased apoptosis of immune effector cells (T cells, B cells, NK cells, neutrophils, dendritic cells) (16, 17), which enables pathogens to evade immune clearance and leads to persistent immunosuppression (9, 18). DAMPs released during the apoptosis of immune cells can induce MODS (19). Animal experiments have confirmed that in the mouse model of sepsis induced by CLP (blindfold ligation and perforation), the apoptosis rate of CD4+ T cells can be as high as 60%, and the surviving cells express high levels of PD-1 (20). Clinical studies and animal models have shown that the peripheral blood and spleen T lymphocytes of sepsis patients/animals show a trend of polarization towards Th2, resulting in an imbalance in the Th1/Th2 ratio. The proportion of Th1 cells in the spleen of CLP mice decreased by 40%, and IL-4 secretion increased by three times, which is significantly correlated with the high mortality rate (19, 21, 22). The imbalance of Th1/Th2 ratio reflects abnormal T lymphocyte differentiation and is accompanied by the dysregulation of the pro-inflammatory/anti-inflammatory cytokine network (23). Dysfunction of monocyte function (such as decreased antigen presentation ability) is one of the important indicators of poor prognosis in sepsis (24, 25).

In the immune suppression induced by sepsis, the excessive expansion and activation of immune regulatory cells, especially regulatory T cells (Tregs) and myeloid-derived suppressor cells (MDSCs), play a crucial role (26, 27). MDSCs are a heterogeneous subgroup of immature myeloid cells that can inhibit the immune response (especially T cells) by mechanisms such as depleting arginine (a T cell essential substance), stimulating the expansion of Tregs, and inhibiting the functions of macrophages and dendritic cells. In sepsis patients, a significant expansion of MDSCs is often detected, and it is associated with an increased risk of secondary infections in critically ill patients (28). Under normal physiological conditions, Tregs account for 5-10% of CD4+ T cells and are crucial for maintaining immune homeostasis and self-tolerance. During sepsis, the frequency of peripheral blood Tregs increases, although the mechanism by which they induce immunosuppression has not been fully elucidated, it is related to the long-term mortality rate of patients (29). In summary, the immune suppression induced by sepsis not only results from the dysfunction and reduction in the quantity of effector immune cells, but is also closely related to the excessive activation and expansion of immune regulatory cells.

#### 2.1.1 Apoptosis

In sepsis, the apoptosis of immune cells (especially lymphocytes) significantly increases. Apoptosis is an important way to remove damaged or aging cells under physiological conditions to maintain homeostasis (30, 31). Apoptosis pathways are of both endogenous and exogenous types. In the exogenous pathway, the Fas/FasL pathway activates Caspase-8, which in turn activates Caspase-3 to initiate apoptosis. The endogenous pathway is regulated by the Bcl-2 family: pro-apoptotic proteins (Bax, Bak, Bim) induce mitochondrial outer membrane permeabilization (MOMP), resulting in the release of cytochrome C and the formation of an apoptotic body with Apaf-1, activating Caspase-9 and Caspase-3, ultimately leading to cell death (32, 33). Studies have shown that in the CLP sepsis mouse model, pro-apoptotic proteins (Bim, cytochrome C) and Caspase-3/9 expression increase, while the expression of anti-apoptotic protein Bcl-2 is suppressed, promoting T cell apoptosis (34). Resendiz-Martinez et al. (35) conducted a comparative analysis between children with sepsis and healthy controls, finding that the levels of apoptosis and Fas expression in peripheral blood mononuclear cells of children with sepsis were significantly increased. The degree of apoptosis was significantly positively correlated with Fas expression. Li et al. (36) confirmed that in the mouse sepsis model established by cecal ligation and puncture, the expression levels of Fas, caspase-3, caspase-8, and caspase-9 were significantly upregulated, while the expression levels of CD4+/CD8+ T lymphocytes and CD19+ B lymphocytes were significantly downregulated. These findings indicate that sepsis enhances immune cell apoptosis through multiple mechanisms, leading to immune deficiency and chronic inflammatory states, increasing the difficulty of treatment and the risk of adverse prognosis.

#### 2.1.2 Pyrolysis

Pyroptosis is a type of programmed cell death triggered by inflammation. Its main characteristic is that cells, upon receiving specific signals or being stimulated, undergo continuous cell swelling, eventually leading to the rupture of the cell membrane, the release of cell contents, and the activation of a strong inflammatory cascade reaction (37). Pyroptosis activation depends on the classical pathway (caspase-1) or the non-classical pathway (caspase-4/5/11) (38–41). The programmed cell death process is usually mediated by inflammasomes, with the most common one being the NLRP3 inflammasome (38). When the inflammasome is activated, it triggers the cleavage and activation of downstream caspase-1 (cysteine aspartic acid protease-1). Activated caspase-1 further cleaves the GSDMD (Gasdermin D) protein, generating a cytotoxic N-terminal fragment (GSDMD-N). The GSDMD-N fragment binds to cell membrane lipids and forms pores on the cell membrane, causing the leakage of cell contents and the release of inflammatory factors, ultimately leading to pyroptosis (39). In patients with sepsis, caspase-1 expression increases. Studies have shown that knocking out caspase-1 can reduce the mortality rate of septic mice (40).

#### 2.1.3 NETosis (a process of forming neutrophil extracellular traps (NETs))

Neutrophil extracellular traps (NETs) are a network structure composed of DNA, histones, and granular proteins released by neutrophils (42). The main function of these traps is to capture and kill invading pathogens, such as bacteria and fungi. The formation process of NETs is called NETosis, which is a special form of cell death, in which neutrophils release their contents into the extracellular environment. When the regulation is imbalanced and leads to excessive release of NETs, it may cause an excessive amplification of the inflammatory response and even trigger immune-related diseases (43). Studies have shown that NETs-induced endothelial injury is a key pathogenic factor of SI-ALI. MSC-EVs can alleviate SI-ALI by inhibiting NETs generation (especially by converting NETosis into apoptosis), providing new theoretical basis and experimental support for the use of MSC-EVs in the treatment of sepsis-related lung injury (44).

#### 2.1.4 Ferroptosis

Ferroptosis is a type of iron-dependent, lipid peroxidation-driven regulated cell death discovered in 2012. It differs from apoptosis, necrosis, pyroptosis, and autophagy in terms of morphological, biochemical, and genetic characteristics (45, 46). Its three core features are: accumulation of redox-active iron, impairment of lipid peroxide clearance, and lipid peroxidation of phospholipids containing polyunsaturated fatty acids (PUFA) (47). Studies have shown that ferroptotic cells release damage-associated molecular patterns (DAMPs), activate inflammatory pathways, and participate in the occurrence and development of organ damage related to sepsis (such as acute kidney injury, liver injury) (48). Glutathione peroxidase 4 (GPX4) inhibits ferroptosis by reducing lipid peroxides and consuming glutathione. Inhibiting GPX4 activity leads to accumulation of lipid peroxides and induces ferroptosis (49). In sepsis, senescent red blood cells rupture and release heme and hemoglobin, which can induce ferroptosis in monocytes and macrophages, and may promote immunosuppression by interfering with signal transduction (such as the STAT1 pathway) (47). Additionally, in the later stage of sepsis, the expression and activity of heme oxygenase-1 (which catalyzes heme degradation) increase. In conclusion, ferroptosis may play an important role in the course of sepsis, especially in organ damage related to septic shock, providing potential new targets for clinical intervention.

#### 2.1.5 Autophagy

Autophagy plays a complex role in sepsis by regulating the immune response and cell survival, exhibiting a “double-edged sword” characteristic (50, 51). Autophagy is a stable process in which cells form autophagosomes to encapsulate and transport damaged proteins, organelles, or pathogens to lysosomes for degradation (52). Moderate autophagy exerts a protective effect in sepsis, with mechanisms including: directly clearing intracellular pathogens (autophagy from the outside), regulating the release of inflammatory factors, promoting antigen presentation, clearing damaged mitochondria (mitochondrial autophagy) to reduce the release of DAMPs, and potential toxin neutralization (53). The function of autophagy is inhibited, which may enhance the phagocytic function of macrophages (52). However, some studies suggest that in specific circumstances (such as excessive activation), inhibiting autophagy may exert a protective effect by alleviating vascular endothelial damage and cytokine storm (54). Autophagy can also regulate the release of inflammatory mediators, enabling the body to be in a relatively immune-tolerant state (55). Therefore, autophagy plays an important but complex role in immune regulation and immunosuppression in sepsis by precisely regulating the inflammatory response, maintaining the function of immune cells, influencing antigen presentation, balancing cell death and metabolism.

### 2.2 Excessive release of anti-inflammatory cytokines

The intense pro-inflammatory response in the early stage of sepsis triggers the body’s negative feedback mechanism to prevent tissue damage caused by excessive inflammation. This process involves the release of various anti-inflammatory cytokines (such as IL-4, IL-10, IL-35, IL-37, IL-38, TGF-β, etc.), aiming to balance the inflammatory response (56). However, overly strong or persistent anti-inflammatory responses directly lead to an immunosuppressive state.

IL-4: Mainly secreted by activated T cells and mast cells. It induces CD4+ T cells to differentiate into Th2 cells, forming a positive feedback loop to promote the production of other anti-inflammatory cytokines, while inhibiting the release of pro-inflammatory cytokines (such as IL-2, IFN-γ), thereby promoting the formation of an anti-inflammatory environment (56). IL-10: A potent immunosuppressive cytokine, mainly secreted by monocytes, macrophages, Th2 cells, and certain Treg subpopulations (57–59). It exacerbates immunosuppression through various mechanisms such as inhibiting the release of pro-inflammatory cytokines (such as TNF-α), inhibiting the proliferation of CD4+ T cells, promoting Treg differentiation, and supporting the expansion of MDSC. In patients with sepsis, the function of IL-10 can be observed, which inhibits T cell proliferation, promotes the production of Treg, and limits the production of effector cytokines (59). IL-35: It is a member of the IL-12 cytokine family, composed of the p35 and Ebi3 subunits, and possesses a strong immunosuppressive function (60). It is mainly produced by regulatory T cells (Tregs, CD4+ Foxp3+ cells) and plays a crucial role in maintaining immune tolerance and suppressing autoimmune responses (61). Studies have found that Tr35 cells have the ability to inhibit T cell proliferation *in vitro*. When Tr35 cells lose IL-35 expression and transform into exTr35 cells, their ability to produce inflammatory cytokines (such as IL-4, IFN-γ, IL-17A) will be restored (58). It can inhibit the proliferation of effector CD4+ T cells, reduce the Th1/Th2 ratio, and inhibit autophagy by restricting the translocation of high mobility group box 1 (HMGB1) (58). IL-37: A new member of the IL-1 family, with significant immunomodulatory and anti-inflammatory effects. It can inhibit the release of pro-inflammatory cytokines by monocytes and neutrophils, as well as regulate the production of anti-inflammatory cytokines. Its expression level is closely related to the severity of immune suppression induced by sepsis (62). Studies have also shown that IL-37 significantly reduces lung edema and cell damage induced by CLP, and simultaneously lowers the levels of inflammatory factors IL-6 and TNF-α, as well as the inflammatory markers PCT and CRP associated with sepsis (63). IL-38: Research has found that the level of IL-38 is elevated in patients with COVID-19 secondary sepsis and is related to the severity of the disease (64). This is consistent with the mechanism of IL-38, which promotes Treg expansion and inhibits the inflammatory response of macrophages (65), suggesting that it may become a new target for immune regulation in sepsis.TGF-β: A multifunctional factor that can induce macrophage polarization to the M2 type, inhibit the proliferation and differentiation of Th1/Th2 cells, promote the apoptosis of immune cells, and lead to immunosuppression (59).

It is important to note that the release of anti-inflammatory cytokines is essentially a protective mechanism of the body against excessive inflammation. However, in the pathological state of sepsis, this anti-inflammatory response becomes out of control and becomes a key factor leading to persistent immunosuppression.

### 2.3 The expression of immune checkpoint molecules increases

Immune checkpoint molecules are key molecules that regulate immune balance and maintain immune tolerance through negative signaling. They mainly include programmed death receptor-1 (PD-1), programmed death receptor ligand-1 (PD-L1), cytotoxic T lymphocyte antigen-4 (CTLA-4), lymphocyte activation gene-3 (LAG-3), T cell immunoglobulin and mucin domain-3 (TIM-3), B and T lymphocyte attenuator (BTLA), etc. (**Table 1**), which are expressed in various immune and non-immune cells. These molecules negatively regulate immune responses by inhibiting the functions of innate immune cells (phagocytosis, pathogen clearance, cytokine release) and inducing T cell exhaustion (functional impairment under continuous antigen stimulation) (67). The upregulation of their expression in sepsis is a core marker of immunosuppression.

**Table 1:** Clinical potential and clinical/eperimental evidence of immune checkpoints in sepsis.

Immune checkpoint	Main potential	Preclinical evidence	Clinical progress	References
PD-1/PD-L1	Restore the function of T cells and reverse immunosuppression	Significantly improved survival rate (animals)	Safety of Phase I/II Trials (No Cytokine Storm)	(66, 67)
CD28	Reduce the inflammatory response and enhance the clearance of bacteria	Significantly reduce mortality rate (animals)	Phase III trial is underway(NCT03403751)	(68)
CD40/CD40L	Reduce cell apoptosis and regulate macrophage function	Combined inhibition of CD80/86 enhances survival rate	A variety of therapeutic agents such as agonists/antagonists monoclonal antibodies, cell vaccines, adenovirus vectors and protein antagonists have been used in early clinical trials for the treatment of malignant tumors, autoimmune diseases and graft rejection reactions.	(69)
OX40L	Inhibit the inflammatory damage mediated by macrophages	The survival rate of OX40L^-^/^-^ mice significantly increased.	The level of expression is related to the mortality rate.	(70)
4-1BB/4-1BBL(CD137L)	Bidirectional regulation (protection of Gram-positive bacteria, inhibition of multiple species)	Improving hippocampal neuroinflammation and behavioral deficits in a mouse model of sepsis-associated encephalopathy	It is necessary to establish specific strategies for the pathogen.	(71)
BTLA	Stage-specific immune regulation	Targeting BTLA may be a new strategy for treating neonatal sepsis.	Dynamic monitoring of expression changes is required.	(72)
Tim-3	Regulating TLR signaling and influencing the balance of cytokines	The mechanism is complex and requires further research.	Tim-3 as an immune status marker	(73)

PD-1/PD-L1: PD-1 is distributed on the surface of activated T cells, B cells, and NK cells. After binding to PD-L1, it recruits the SHP2 phosphatase, inhibits the downstream signal of TCR, and leads to the inhibition of T cell proliferation, reduced cytokine secretion, inactivation, and apoptosis (74). The enhanced expression of PD-1 on peripheral blood T cells of patients with sepsis is associated with weakened T cell function, increased hospital infection rate, and mortality. In the CLP model, PD-1 on CD4+ T cells is upregulated within 24 hours, PD-1 on CD8+ T cells is upregulated within 3–7 days, and PD-L1 on myeloid cells is rapidly upregulated within 24 hours, jointly inhibiting the ability of T cells to clear pathogens (74). Clinical studies have confirmed that the expression of PD-1 in immune cells of sepsis patients is upregulated, accompanied by the downregulation of HLA-DR and CD28 and the increase in Treg activation (75), directly correlating the PD-1 pathway with poor prognosis.CTLA-4: Highly homologous to CD28, it competitively binds to CD80/CD86, interrupting the CD28 co-stimulatory signal, and transmitting inhibitory signals to inactivate T cells (76). The expression of CTLA-4 on CD4+ T cells of sepsis patients is significantly increased, suggesting it as a potential therapeutic target (77). TIM-3: The expression of TIM-3 on CD4+ T cells of sepsis patients increases (78). TIM-3 negatively regulates T cell proliferation and effector function by binding to ligands (such as galectin-9) or interacting with MHC II molecules.BTLA: Belongs to the immunoglobulin superfamily, it can inhibit T cell activation, induce Treg immune tolerance, promote IL-4 expression, and inhibit IL-17 and TGF-β1 expression (13). The level of soluble BTLA in sepsis patients continuously increases and is significantly associated with increased risk of death at 90 days and 1 year (79).

In summary, the upregulation of immune checkpoint molecule expression is one of the core mechanisms of immune suppression in sepsis. Further exploration of its dynamic changes characteristics at different stages of sepsis will help clarify new therapeutic targets.

### 2.4 Immunocyte metabolic disorder

“Cytokine storm” and “genomic storm” can be used to describe the “systemic inflammatory response” in the early/acute stage of sepsis, but these are oversimplifications of the innate and adaptive immune responses (80). The immune imbalance in sepsis can develop into the “persistent inflammation-immunosuppression-catabolism syndrome” (Persistent Inflammation, Immunosuppression, and Catabolism Syndrome, PICS) characterized by persistent inflammation, immunosuppression, and catabolism (81–83). The role of metabolic reprogramming of immune cells in regulating their functional state is increasingly being recognized. The metabolic dysregulation of immune cells (mitochondrial dysfunction, enhanced glycolysis, and inhibited oxidative phosphorylation) in sepsis is a key factor leading to cell dysfunction, decreased pathogen clearance ability, and the formation of an immunosuppressive microenvironment (9, 84, 85).

The functions of immune cells are regulated by their energy states. Resting immune cells mainly rely on oxidative phosphorylation (OXPHOS) to produce ATP. Upon activation (such as in response to pathogens), metabolic reprogramming occurs to meet the rapid energy demands and biosynthetic needs, manifested as enhanced glycolysis (the Warburg effect), with glycolysis being prioritized even in the presence of oxygen, often accompanied by inhibition of OXPHOS or mitochondrial dysfunction (86–88). This helps with the early rapid response of immune cells. However, in the persistent state of sepsis, this reprogramming is dysregulated. Long-term reliance on glycolysis not only results in low ATP production, leading to depletion of ATP and NAD+, but also causes a large accumulation of lactic acid and acid-base imbalance (87, 89). The high lactic acid environment has immunosuppressive effects and can lead to tissue damage (84). Studies have shown that in the early stage of excessive inflammation in sepsis, the expression of glycolysis-related genes and key regulatory molecules (mammalian target of rapamycin, mTOR, hypoxia inducible factor-1α, HIF-1α) in peripheral blood mononuclear cells is significantly upregulated. In M1-type macrophages, the expression of mTOR and HIF-1α increases, and enhanced glycolysis supports their rapid production of ROS and pro-inflammatory cytokines to kill pathogens (89). Pro-inflammatory T cell subsets (Th1, Th2, Th17) also show enhanced glycolysis. Moreover, the metabolic changes in sepsis (changes in oxygen consumption, abnormal substrate cycling, ketogenic disorder, amino acid metabolism disorder, lipid oxidation impairment, mitochondrial dysfunction) are all associated with organ dysfunction and poor prognosis (84). The functional defects caused by metabolic disorders of immune cells not only hinder the clearance of primary infections but also increase the risk of latent virus reactivation (such as CMV, EBV) and the risk of long-term secondary infections and poor prognosis (90).

### 2.5 Persistent inflammatory response

The immune suppression induced by sepsis is intertwined with persistent inflammatory responses, activation of the coagulation system, vascular endothelial damage, dysregulation of the complement system, and disruption of the intestinal microbiota, forming a complex heterogeneous syndrome (91). Immune damage drives the interaction of white blood cells, cytokines, oxygen free radicals, endothelial cells, complement and coagulation systems, resulting in a persistent inflammatory response and additional tissue damage (92). The massive release of pro-inflammatory factors such as TNF-α and IL-1β induces an inflammatory factor storm (93). Activated neutrophils release reactive oxygen species and proteases, exacerbating tissue damage. The neutrophil extracellular traps (NETs) both defend and can cause tissue damage (94). The release of potent pro-inflammatory molecules such as C3a and C5a from the complement system recruits activated immune cells and platelets, increases vascular permeability, promotes white blood cell adhesion and migration, and leads to vascular damage (95). The inflammatory damage to the vascular endothelium disrupts the glycocalyx, activates pro-coagulant activity, and simultaneously damages the endogenous anticoagulation mechanisms (antithrombin, tissue factor pathway inhibitor, protein C system), resulting in coagulation dysfunction (96). These dysregulated immune responses and cascade effects ultimately lead to severe multi-organ dysfunction in the central nervous system, kidneys, blood, liver, respiratory, and cardiovascular systems, significantly increasing the risk of death.

### 2.6 Reduced expression of human leukocyte DR antigen

The immunosuppression associated with sepsis is accompanied by excessive inflammatory responses, damage to immune cells, metabolic disorders, increased release of anti-inflammatory cytokines, and direct effects of pathogen-associated molecular patterns. These mechanisms work together to weaken the function of antigen-presenting cells, thereby affecting the immune response capacity of the body. The impaired function of antigen-presenting cells (APC) is an important feature of immunosuppression in sepsis, and the downregulation of human leukocyte antigen DR (HLA-DR) expression on the surface of monocytes is a key indicator (85, 90), which is one of the important factors contributing to poor prognosis in sepsis (24, 25). HLA-DR is a major histocompatibility complex II (MHC-II) molecule that is crucial for activating CD4+ T cells-mediated adaptive immunity. Its expression level is an important indicator for evaluating the functional status of APC.

Stimuli related to sepsis (such as LPS) can down-regulate the expression of HLA-DR in monocytes, and the mechanism involves abnormal regulation of HLA-DR transcription (such as CIITA, IRF1) and signaling pathways (such as c-Src, Stat1 phosphorylation) (96). The reduction of HLA-DR expression in bone marrow monocytes is closely related to the clinical prognosis of sepsis (97), and it is negatively correlated with the Sequential Organ Failure Assessment (SOFA) score (98). The proportion of HLA-DR-positive monocytes below 30% is regarded as an indicator of immunosuppression (99). Studies have found that the level of mHLA-DR in the early stage of the disease course (1–8 days) of deceased sepsis patients is significantly lower than that of survivors, and the degree of mHLA-DR inhibition is positively correlated with the rate of secondary infection (100). Therefore, dynamic monitoring of the expression level of mHLA-DR is an important biomarker for evaluating the immune status of sepsis patients, predicting the risk of secondary infection and prognosis (101).

## 3 Therapeutic strategies for immunosuppression in sepsis

### 3.1 Immune stimulating cytokines

Granulocyte-macrophage colony stimulating factor (GM-CSF) (102) is a hematopoietic growth factor that can promote the generation of neutrophils, monocytes, and macrophages, and enhance the expression of human leukocyte antigen-DR (HLA-DR) on monocytes. An *in vivo* and *in vitro* study (103) demonstrated that recombinant GM-CSF can increase the expression of IL-1β in polymorphonuclear neutrophils, thereby enhancing their bactericidal ability and improving the prognosis of secondary Pseudomonas aeruginosa pneumonia induced by sepsis. GM-CSF treatment has been proven to significantly reduce the levels of bone marrow-derived suppressor cells (MDSCs) and regulatory T cells (Tregs) in sepsis patients, enhance the function of CD4+ T cells, improve the immune status, and thereby reduce the risk of infection and improve clinical prognosis. Interleukin-7 (IL-7) is an important cytokine involved in immune regulation and enhancement. It has strong anti-apoptotic properties and promotes the proliferation of T lymphocytes. Additionally, IL-7 can down-regulate the expression of TGF-β and inhibit the immunosuppressive mechanism, thus having important application potential in anti-tumor immunotherapy. An animal experimental study (104) showed that MVA-hIL-7-fc is a construct that uses the modified vaccinia Ankara (MVA) strain as a vector to deliver human interleukin-7 (IL-7), which can effectively activate the innate and adaptive immune systems. In mouse models, this treatment regimen increased the number and functional activity of B cells, T cells, natural killer cells (NK cells), and myeloid-derived suppressor cells (MDSCs), and significantly improved the survival rate of models of cecal ligation and puncture (CLP) and white candida-induced sepsis. MVA-hIL-7-fc is expected to become a new type of immunotherapy agent, although further verification is needed. Interleukin-15 (IL-15) is an immunoregulatory cytokine that can promote the development and activation of natural killer cells (NK cells). Animal studies have shown that IL-15 is up-regulated in myocardial cells of sepsis mice (105). Exogenous IL-15 has been proven to alleviate inflammatory responses, improve cardiac function, possibly by down-regulating the expression of caspase-3 and Bax, up-regulating the expression of Bcl-2, inhibiting the NF-κB signaling pathway, and thereby reducing cardiomyocyte apoptosis. Another study has shown that subcutaneous injection of IL-15 can reduce T cell apoptosis in sepsis mice, increase the number and functional activity of NK cells and macrophages, and improve survival rates (106). However, the potential mechanism by which IL-15 reverses T cell exhaustion still needs to be further elucidated.

### 3.2 Immune checkpoint inhibitors

PD-1/PD-L1 antibodies include well-known examples such as nivolumab, pembrolizumab, and atezolizumab. Hotchkiss et al. (67) and their colleagues evaluated the clinical safety and pharmacokinetics of the anti-PD-1 antibody nivolumab in patients with sepsis. The study demonstrated that nivolumab showed good safety, providing a crucial foundation for the initiation of subsequent clinical trials evaluating anti-PD-1 antibody treatment for sepsis in large, international, and multicenter cohorts. An animal study showed that anti-PD-L1 antibody treatment significantly reduced the levels of inflammatory cytokines in the serum of septic mice, including IL-6, TNF-α, and IL-10, while increasing the survival rate of the animals (107). The anti-PD-L1 nanobody KN035 (108) has been proven to reverse immune cell apoptosis, inhibit bacterial proliferation, and significantly increase the 7-day survival rate of septic mice. PD-1/PD-L1 inhibitors have shown significant efficacy in immunotherapy for malignant tumors. Therefore, anti-PD-1/PD-L1 treatment is a promising therapeutic strategy for severely septic patients with significant immunosuppression, and its efficacy and safety merit further verification through large-scale clinical trials. The expression of T-cell surface CTLA-4 is significantly upregulated in septic patients. Both *in vitro* and *in vivo* studies (109) have shown that CTLA-4 antibody treatment can significantly increase the cumulative survival rate of septic mice for 96 hours, and its potential mechanism may be related to promoting the expression of CD69 on T lymphocytes and enhancing T cell activation. Mouse experiments have shown (110) that in the sepsis model characterized by chronic alcohol intake, the increase in CTLA-4 expression is associated with poorer clinical outcomes. Notably, CTLA-4 inhibitor treatment specifically provided survival benefits in this subgroup of mice, suggesting a potential strategy for precise treatment of alcohol-related sepsis. The TIM-3 antibody is another promising immunotherapy approach because the expression of TIM-3 on T cells of septic patients is upregulated and is associated with systemic immunosuppression and poor prognosis. Liu et al. (111) demonstrated that anti-TIM-3 antibody treatment can alleviate multi-organ damage caused by sepsis and effectively regulate excessive inflammatory responses. α-Lactose, as an inhibitor of the TIM-3 signaling pathway, has been proven to inhibit the *in vitro* apoptosis of NK cells in septic patients (112). Although preclinical studies have shown the therapeutic potential of targeting TIM-3 in sepsis, high-quality clinical evidence regarding the application of TIM-3 antibodies in septic patients is still limited. Monotherapy targeting TIM-3 may be associated with suboptimal efficacy, highlighting the urgent need to study novel combined strategies that combine TIM-3 blockade with other immune pathway regulation.

### 3.3 Mesenchymal stem cells

Mesenchymal stem cells (MSCs) alleviate organ damage by enhancing bacterial clearance, regulating immune responses, inhibiting cell apoptosis, and promoting tissue repair, thereby reducing the mortality rate associated with sepsis. Studies have shown (113) that MSCs promote the differentiation of M2 macrophages, enhance phagocytic activity, alleviate immunosuppression, and thereby promote tissue repair. In cases of sepsis progression, MSCs exhibit prolonged retention in damaged organs, which is related to the reduction of iron-induced damage in macrophages (114). Alp et al. (115) reported that in a clinical study involving 10 patients with septic shock, the treatment group that received MSC infusion had a survival rate of 100% on the 14th day, significantly higher than the 70% survival rate of the control group (p < 0.05). Additionally, the improvement in the Sequential Organ Failure Assessment (SOFA) score in the treatment group was also significantly greater than that in the control group. These findings suggest that MSCs may increase the early survival rate of patients with sepsis. However, due to the limited sample size, further verification is needed through large-scale randomized controlled trials. In *in vivo* and *in vitro* studies (116), CD146+ mesenchymal stem cells increase the proportion of specific immune cell populations, enhance macrophage phagocytosis, expand the pool of reparative macrophages, promote the secretion of vascular endothelial growth factor (VEGF), rapidly inhibit inflammatory responses, improve the organ pathology of septic animals, and ultimately lead to a significant increase in survival rate. In the mouse sepsis model (117), extracellular vesicles derived from mesenchymal stem cells (MSCs) combined with azithromycin significantly alleviated the symptoms of the animals, providing preliminary evidence for potential clinical translation, although further verification is needed. A meta-analysis of 20 preclinical comparative studies on sepsis models indicated that MSC treatment could reduce the mortality rate of experimental sepsis by approximately 73% (118). Increasing evidence supports the therapeutic potential of MSCs in sepsis. Currently, two phase II randomized controlled trials are evaluating their effects on immune regulation and organ dysfunction in patients with septic shock.

### 3.4 Calcium protective protein inhibitor

The calcium protection protein is a heterodimer formed by two calcium-binding proteins, S100A8 and S100A9. It is mainly secreted by neutrophils and monocytes and is closely related to the activity and severity of sepsis. Under sepsis conditions, the calcium protection protein not only promotes the release of pro-inflammatory cytokines, thereby amplifying the inflammatory cascade reaction, but also interacts with the toll-like receptor 4 (TLR4) on dendritic cells (DC) precursor cells, contributing to immunosuppressive effects (119). Experimental evidence (120) indicates that administering the calcium protection protein inhibitor, pakinimod, can significantly improve renal dysfunction and histopathological changes in sepsis mice, while partially restoring sepsis-related immunosuppression.

### 3.5 TREM-1 inhibitor

Trigger receptor-1 (TREM-1) expressed on myeloid cells plays a key regulatory role in the inflammatory cascade reaction during sepsis. It is a myeloid cell-specific activating receptor, mainly expressed on the surface of neutrophils, monocytes, and macrophages. The activation of TREM-1 triggers the production and release of pro-inflammatory cytokines and chemokines, thereby amplifying systemic inflammation. Additionally, it promotes the release of neutrophil extracellular traps (NETs), which helps in the subsequent development of endothelial damage and vascular dysfunction in sepsis (121). LP17 is a TREM-1 blocking peptide that has been proven to reduce cytokine secretion in macrophages and improve hemodynamic parameters and survival rates in LPS-induced sepsis mouse models (122). LR12 (nangibotide) (123) is a 12-amino acid peptide derived from trem-like transcript-1 (TLT-1), being the first TREM-1 inhibitor to enter clinical trials, marking an important step towards translational applications. Francois et al. (124, 125) reported that nangibotide did not reach the primary endpoint in the 2a/2b phase clinical trial; however, in patients with septic shock with elevated plasma sTREM-1 levels, nangibotide significantly improved the SOFA score on the 5th day. It is notable that the high-dose group showed more significant effects, indicating a dose-dependent relationship. Other TREM-1 inhibitors are currently under development, and further research is needed to determine the patient population most likely to benefit from this adjunctive treatment strategy.At present, in both preclinical and clinical studies, there are many new treatment options (**Table 2**).

**Table 2:** Emerging therapies for immune therapy in sepsis.

Immunotherapy	Cellular pathways/biomarkers	Therapeutic target/mechanism	Significance	Restriction/current verification	References
Preventive treatment with human amniotic stem cells (hAFSCs)	CD90、CD73和CD105	Inhibited the acute reactive gliosis and reduced the proliferation of astrocytes in the hippocampus.	Improved the spatial perception and memory-based behavioral abilities during adolescence in neonatal rat survivors with sepsis	Newborn rat model of sepsis	(126)
MSCs and the MSC secretome improve organ dysfunction caused by sepsis	MSC-1, MSC-2	IL-6、TNF-α、IFN-γ、 JAK-1、 JAK-2、 STAT-3、 SOCS-3、IL-10	MSCs significantly improve organ function in sepsis through multi-target immune regulation (anti-inflammatory, antibacterial, and promoting repair), and reduce mortality.	Technical challenges in clinical translation (such as cell stability, drug delivery safety)	(127)
Nanoparticles (NPs): The application of nano carriers, bio-inspired nanoparticles and self-assembled nanomaterials		Disrupting protein functions, physically damaging cell structures, generating reactive oxygen species, depleting antioxidants, damaging cell membranes, interfering with nutrient absorption and dephosphorylating tyrosine residues, etc.	Precise drug therapy, reduction of side effects caused by drugs, improvement of treatment efficacy, as well as efficient diagnosis and treatment of sepsis	Lack of large-scale clinical trials, focus on the safety and feasibility of clinical trials	(128)
Dual-function antibiotic adjuvant	A surfactant with a cyclic hydrophobic side group and two or three amine groups (NNaph, NDiphe and NAda)		D-LBDiphe exhibits a multi-modal mechanism of action, enhancing the efficacy of antibiotics by approximately 4100 times.	Lack of *in vivo* research to verify its potential as a clinical candidate drug	(129)

## 4 Diagnostic value of key biomarkers

### 4.1 HLA-DR

Patients with severe conditions often have early acquired immune suppression (AIDs), but the immune suppression characteristics of patients with different causes (such as sepsis, nerve injury, trauma, or postoperative conditions) may vary. All patients may show early downregulation of mHLA-DR, but persistent low expression or a downward trend indicates a significant correlation with the risk of ICU-acquired infections (IAI) in patients with sepsis (130). The expression kinetics of HLA-DR detected by flow cytometry is related to prognosis, but the infection site or pathogen type is not significantly associated with the kinetics of HLA-DR (24, 131). Some studies have combined the expression of mHLA-DR and the release of TNF-α *in vitro* to identify patients at high risk of immune suppression, and the results showed that these two biomarkers were related to adverse clinical outcomes (death or secondary infection) (132). The immune status of patients with sepsis is particularly important in precision medicine. Studies have shown that the continuous low expression of mHLA-DR (especially continuous < 5000 AB/c) is a reliable indicator for predicting IC (invasive Candida infection). In patients with septic shock, monitoring the immune status by detecting the dynamic changes of mHLA-DR to optimize antifungal treatment is particularly important (133). However, current research is mainly limited to small sample single-center studies, with high detection requirements. Therefore, the research conclusions may not have significant impact. The popularization of flow cytometry detection technology for mHLA-DR may be combined with current cytokine detection and pathogenological examination to better predict the immune suppression status of sepsis patients and better guide clinical treatment.

### 4.2 PD-1/PD-L1

Myeloid-derived suppressor cells (MDSCs) participate in the immune suppression induced by sepsis through the PD-L1/PD-1 axis (74). This discovery provides a target for immune regulation therapy, reversing the immune suppression state, and also offers an immune monitoring marker for the early detection of the immune suppression state in sepsis patients. The research results revealed that the SOFA score combined with the predictive model of SOFA + NK PD-L1 (a model that integrates SOFA score and the percentage of PD-L1+ natural killer (NK) cells) demonstrated significantly superior performance in predicting the 28-day mortality risk of sepsis patients (134). In the future, through large-sample multicenter studies, the model can be optimized by dynamic monitoring of PD-L1+ NK cells, and precise targeted immunotherapy strategies targeting the PD-1/PD-L1 pathway can be explored. Preclinical research results showed that anti-PD-L1 antibodies significantly improved the survival outcome of the burn infection model by reversing immune suppression and enhancing the antibacterial response, providing a new strategy for clinical treatment of burn-related infections (135). However, the research conclusions need to be further verified in clinical practice, especially for sepsis patients, and whether the combination of “enhancing immunity + direct killing” through “enhancing immunity + direct killing” can further improve clinical efficacy when used in conjunction with antibiotics. Some studies have used rapid SaO_2_ assessment at admission to screen out sepsis patients with high PD-L1 expression and guide anti-PD-1 immunotherapy to avoid ineffective treatment and improve prognosis (136). This research conclusion also provides a new strategy for precise immunotherapy of sepsis patients, through clinical detection indicators for initial clinical screening, and monitoring of immune markers for high-risk groups to guide clinical treatment.

### 4.3 sTREM-1

sTREM-1 is an amplifier of the innate immune response. Its soluble form can be detected in various body fluids, making it a potential alternative marker for immune activation in patients with sepsis. Studies have explored the diagnostic value of sTREM-1 for systemic or local infections. The results showed that due to the fact that sTREM-1 is susceptible to non-infectious inflammation (such as trauma, autoimmune diseases, cardiovascular diseases), it has high sensitivity and specificity in local infection sites (such as BALF, pleural effusion, ascites), and can significantly distinguish between infected and non-infected states (137). Perhaps sTREM-1 can serve as a marker for monitoring the therapeutic effect of local infections. During the treatment of critically ill internal medicine patients, the condition may worsen and lead to sepsis, so it is particularly important to find early indicators for sepsis. Multi-center research results have shown that sTREM-1 and presepsin can be potential biomarkers for the early diagnosis of acute-on-chronic liver failure (ACLF) patients with sepsis, and the diagnostic method combining presepsin with CLIF-SOFA score has good application prospects (138). Sepsis is an important cause of morbidity and mortality in newborns, and early detection and intervention are particularly important. It has been found that sTREM-1 can be used for the early diagnosis of neonatal sepsis (139). sTREM-1 can not only be used as a diagnostic indicator but also as a predictive indicator for the prognosis of sepsis patients. A prospective study evaluated multiple biomarkers and showed that sTREM-1 can independently effectively predict the mortality rate of patients with infections in tropical regions, and combining with clinical variables can further improve the prediction accuracy (140).

### 4.4 The role of emerging technologies

The traditional blood culture testing method is limited in diagnosing sepsis due to the influence of drugs and the timing of testing. The emerging molecular diagnostic technologies have demonstrated significant advantages in terms of sensitivity, sample volume, detection of multi-microbial infections, and antimicrobial resistance, and can quickly and accurately diagnose sepsis. Therefore, they show significant advantages in clinical practicability. These include nucleic acid amplification techniques (NAATs) (Iridica Plex ID, SeptiFast, SepsiTest, U-dHRM, MinION nanopore sequencing), host response-targeted techniques (SeptiCyte Lab), non-amplification techniques (IC³D), and machine learning-assisted algorithms (InSight, TREWScore) (141–149). Additionally, the integration analysis of metabolomics and transcriptomics (RNA sequencing) can be utilized to identify specific biomarkers in sepsis patients as new targets for diagnosis and treatment. Studies have found that core genes ITGAM and C3AR1 are expressed at higher levels in the plasma of sepsis patients (150), and through clinical translation, they can become new targets for the diagnosis and treatment of sepsis. Currently, the main detection techniques used in intensive care units are metagenomic RNA and DNA sequencing technologies, which can quickly and accurately identify pathogens and guide anti-infection treatment (151). In our previous research, we also found that metagenomic sequencing technology is not only used to guide treatment, detect pathogenic microorganisms early, intervene early, but also improve the prognosis of sepsis patients (6).

## 5 Consistency and heterogeneity of mechanisms and treatment strategies

The pathogenesis of sepsis is a dynamic process that evolves over time. During the acute phase of the disease and the subsequent immunosuppressive phase, different clinical manifestations will be observed, and some immune mechanisms remain controversial. Moreover, in animal models, *in vitro* experiments, and clinical trials, immune checkpoints and therapeutic effects will have different manifestations. Therefore, it is particularly important to identify the consistency and heterogeneity of immune mechanisms and immunotherapy strategies. The conclusions of some preclinical studies and early clinical trials on certain pathways have shown promising clinical prospects. For example, multiple research groups have tested IL-7 in animal models of sepsis, which can prevent lymphocyte apoptosis, restore the functions of CD4+ and CD8+ T cells, and improve survival rates (152). Clinical trials have shown that CYT107 reversed the significant reduction in CD4+ and CD8+ immune effector cells, representing a new treatment method for patients with sepsis (153). At the same time, it has been verified that CYT107 has good tolerance and no evidence suggests that it causes cytokine storms or aggravates inflammation or organ dysfunction.

Although a large number of *in vitro* experiments and some clinical trials have characterized the immunotherapy strategies for sepsis, there are still some pathways that are contradictory in terms of translational relevance. In animal models and *in vitro* analyses, immunorecovery effects have been demonstrated, but clinical trials in sepsis patients are still limited, and the efficacy may show significant differences depending on the disease stage and phenotype, and may also lead to immune-related complications.

NETosis: *In vitro* experiments and mouse models have revealed the role of neutrophil extracellular traps (NETs) in sepsis. Using recombinant DNase or PAD-4 inhibitors can treat sepsis and alleviate the severity of the disease. Currently, due to the safety data of existing drugs supporting it, rhDNase combined with antibiotics is prioritized for pediatric Phase II clinical trials, but the combined treatment plan needs to be strictly designed and the risk of bleeding needs to be monitored. PAD-4 inhibitors are advanced to adult Phase I trials first, and after accumulating safety data, they are expanded to pediatric use, with a focus on developing new highly selective inhibitors (154).

In preclinical studies, anti-PD-L1 antibodies (such as Atezolizumab) significantly improve the survival rate of septic mice and reverse immune cell dysfunction (66), while the nanobody KN035 simultaneously improves lung, liver, and spleen damage and reduces the levels of inflammatory factors (108). High-dose (960mg) Nivolumab (anti-PD-1 antibody) may cause long-term immunosuppression (>90 days), and may also cause immune-related adverse reactions (irAEs): such as pneumonia, thyroiditis, which require close monitoring (155).

## 6 Limitations of clinical translation

### 6.1 Patient heterogeneity and precise stratification

Question: The causes of clinical sepsis (bacterial/viral/fungal), age, and underlying chronic diseases (endocrine system diseases, cardiovascular system diseases, immune-related diseases) vary greatly. The phenotypes of sepsis also differ significantly, which may mask the true therapeutic effect. Failure to precisely select the target patients may lead to negative results.
Proposed solution: Precisely select biomarkers, choose screening tools with good specificity; stratify patients by age, pathogen, and degree of organ failure (using APACHE II score or SOFA score) for subgroup analysis.

### 6.2 Safety risk

Hemorrhagic tendency: Patients with sepsis often have coagulation dysfunction. The degradation of DNA by DNase may disrupt the fibrin network, increasing the risk of bleeding (especially intracranial hemorrhage). For example, the use of rhDNase in cystic fibrosis has been reported to cause hemoptysis as a side effect, but the coagulation function of patients with sepsis is more fragile.
Immunomodulatory imbalance: NETs have antibacterial effects, and excessive clearance may weaken the clearance of pathogens.
Off-target effect: PAD-4 is involved in the citrullination of various proteins (such as keratin and fibrinogen). Long-term inhibition may affect wound healing, coagulation function, or autoimmune responses.
Developmental specificity toxicity: The infant’s enzyme system is immature, and the pharmacokinetic (PK/PD) data is lacking, making it prone to accumulation and poisoning.

### 6.3 The challenge of optimizing the drug administration plan

Timing and dosage: In animal models, repeated administration was carried out every 12 hours. However, in clinical scenarios, patients may have a delay in seeking medical attention (such as several hours later), thus missing the optimal intervention window.
Dosage conversion is complex: it needs to be based on body weight or body surface area, and there is a significant variation in the drug distribution volume among infants.
Administration route: The animal model part is based on intraperitoneal injection as the foundation, but in patients with sepsis, intravenous administration is more common, and the bioavailability is unknown.

### 6.4 Dispute over the endpoint for evaluating therapeutic efficacy

In animal models, mortality or survival rate is used as the endpoint for evaluating therapeutic efficacy. However, in clinical practice, the focus should be on organ function recovery, enabling patients to achieve the maximum clinical benefit. Moreover, the long-term effects are unknown, and in the future, animal models may need to be designed for long-term follow-up (such as 6 months to 1 year).

### 6.5 Technical and cost limitations

High-precision and high-cost methods (such as the PicoGreen method) have not yet been widely used in clinical practice. Central laboratories need to be established for unified testing. Biologics (such as dornase alfa) are expensive, and there are no mature drugs for PAD-4 inhibitors, with high development costs.

## 7 Limitations

This study provides a comprehensive review of the immune mechanisms and treatment strategies of sepsis. Unlike systematic reviews, comprehensive reviews do not follow a standardized framework. They mainly conduct a broad synthesis of existing evidence, although the scope of inclusion is wide, but it has limitations. Due to the lack of strict systematic methods in narrative reviews, it increases selection bias and may lead to the omission of some research results. Secondly, the lack of comparability between preclinical studies and clinical studies limits their translational value. Nevertheless, this review mainly focuses on the breadth of research results rather than their rigor, and no formal bias risk assessment or meta-analysis was conducted. Despite these limitations, this review provides a latest comprehensive perspective on linking molecular mechanisms, clinical significance, and treatment strategies, in order to promote the development of immunotherapy strategies for sepsis.

## 8 Summary and prospects

In summary, the immunosuppression induced by sepsis activates the immune response to pathogens and PAMPs, leading to metabolic disorders and oxidative stress, causing damage and apoptosis of immune cells. The persistent inflammatory response and the increase in anti-inflammatory factors attempt to balance the excessive immune response, but instead result in the inhibition of the immune response. In the immunosuppression of sepsis, the expression of antigen-presenting molecules such as HLA-DR decreases, while the expression of immune checkpoint molecules increases, disrupting the adaptive immune response. Ferroptosis and autophagy play a role in responding to cellular stress and damage, but dysregulation of autophagy may exacerbate the loss of immune cell function. All these mechanisms interact with each other, jointly leading to sepsis-related immunosuppression, increasing the risk of infection and multiple organ dysfunction.

Although the treatment of sepsis still faces significant challenges, a comprehensive understanding of its immunosuppressive mechanism undoubtedly opens up new perspectives for developing new therapeutic strategies. Major breakthroughs have been made in the field of immunosuppressive treatment for sepsis, and three cutting-edge treatment strategies have shown great potential for clinical translation. In the field of targeted cytokine therapy, granulocyte-macrophage colony-stimulating factor (GM-CSF) works in synergy with interleukin-7 (IL-7), which has dual effects of inhibiting cell apoptosis and promoting T cell proliferation, and interleukin-15 (IL-15), which specifically activates natural killer cells (NK cells), to form a multifunctional treatment plan based on cytokines. In the field of reprogramming of immune checkpoints, PD-1/PD-L1 inhibitors dynamically regulate the inflammatory response and synergistically supplement CTLA-4 and TIM-3 antibodies, establishing a new paradigm of precise immunotherapy. The most breakthrough progress is the new regulatory strategies, including mesenchymal stem cell (MSC) therapy, which has been proven to reduce mortality by up to 73%; calcium protectin inhibitor can precisely regulate the immune microenvironment; and TREM-1 inhibitor, which can rapidly alleviate the inflammatory response. These methods together constitute a multi-targeted treatment system. Through the synergistic mechanism of immune reconstruction, checkpoint regulation, and microenvironment regulation, these innovative therapies provide a paradigm shift strategy for reversing immune paralysis in sepsis.

The future research focus should be concentrated on the following aspects: First, conduct in-depth analysis of the functional changes and interaction mechanisms of different immune cells in sepsis; second, explore the regulatory mechanism of the cytokine network in sepsis, identify key regulatory nodes and targets; third, deeply study the interactions among multiple immune checkpoints in immune suppression induced by sepsis, and develop new immunotherapy strategies; fourth, further investigate the dual roles of cell death and survival pathways such as ferroptosis and autophagy in sepsis, providing a theoretical basis for precise intervention. In summary, the immune suppression mechanisms caused by sepsis are complex and diverse. A comprehensive understanding and precise regulation of these mechanisms will help reduce the long-term mortality rate associated with sepsis and improve patient prognosis. Through the integration of multidisciplinary research results and the development of targeted immunomodulatory treatment strategies, new hope is expected to be brought to sepsis patients in the future.
